# Epigenetic perspective on the role of brain-derived neurotrophic factor in burnout

**DOI:** 10.1038/s41398-020-01037-4

**Published:** 2020-10-19

**Authors:** Jelena Bakusic, Manosij Ghosh, Andrea Polli, Bram Bekaert, Wilmar Schaufeli, Stephan Claes, Lode Godderis

**Affiliations:** 1 Centre for Environment and Health, Department of Public Health and Primary Care, KU Leuven, Leuven, Belgium; 2 Pain in Motion research group (PAIN), Department of Physiotherapy, Human Physiology and Anatomy, Vrije Universiteit Brussel, Brussels, Belgium; 3 Department of Forensic Medicine, Laboratory of Forensic Genetics and Molecular Archaeology; KU Leuven, Leuven, Belgium; 4 Work, Organisational and Personnel Psychology, KU Leuven, Leuven, Belgium; 5 Psychiatry Research Group, Department of Neuroscience, KU Leuven, Leuven, Belgium; 6 IDEWE, External Service for Prevention and Protection at Work, Heverlee, Belgium

**Keywords:** Diagnostic markers, Clinical genetics, Epigenetics and plasticity, Clinical genetics

## Abstract

Brain-derived neurotrophic factor (BDNF) plays a potential role in the neurobiology of burnout, but there are no studies investigating the underlying genetic and epigenetic mechanisms. Our aim is to further explore the role of BDNF in burnout, by focusing on the Val66Met polymorphism and methylation patterns of the *BDNF* gene and serum BDNF (sBDNF) protein expression. We conducted a cross-sectional study by recruiting 129 individuals (59 with burnout and 70 healthy controls). Participants underwent a clinical interview, psychological assessment and blood sample collection. Polymorphism and DNA methylation were measured on DNA from whole blood, using pyrosequencing and sBDNF levels were measured using ELISA. We found significantly increased methylation of promoter I and IV in the burnout group, which also correlated with burnout symptoms. In addition, DNA methylation of promoter I had a significant negative effect on sBDNF. For DNA methylation of exon IX, we did not find a significant difference between the groups, nor associations with sBDNF. The Val66Met polymorphism neither differed between groups, nor was it associated with sBDNF levels. Finally, we did not observe differences in sBDNF level between the groups. Interestingly, we observed a significant negative association between depressive symptoms and sBDNF levels. The current study is the first to show that *BDNF* DNA methylation changes might play an important role in downregulation of the BDNF protein levels in burnout. The presence of depressive symptoms might have an additional impact on these changes.

## Background

Today, burnout has become one of the most widely discussed mental health problems in the workplace. With increasing job demands and time pressure, workers in many sectors and industries suffer from severe fatigue, which comprises of physical, mental, emotional and behavioural symptoms –which is commonly referred to as job burnout^1^. According to recent research conducted in Belgium, more than 7% of the working population have burnout complaints, whereas another 9% are “at risk” of developing burnout^2^. European data show similar numbers, indicating that on average 10% of the EU workforce feels burned-out^3^. Despite the recognition of burnout as a societal problem worthy of attention, there is still a debate among researchers and practitioners about what burnout really is, what symptoms are associated with it, and whether it can be considered as a distinct mental disorder, especially relative to depression^4^. According to the most widely used definition, burnout refers to a three-dimensional syndrome composed of exhaustion, cynicism or a negative attitude towards work, and reduced professional efficacy^5^. This definition has also been included in the updated version of ICD-11^6^, where burnout has been classified as an occupational phenomenon. Still, no uniform diagnostic guidelines have been developed.

A recent literature review^1^ shows that burnout research mainly focused on causes and associated factors, whereas studies on biological correlates are the least represented. In addition, the biological studies on burnout are heterogeneous in design, burnout assessment and laboratory techniques to quantify biomarkers, which make the comparison inconclusive^7^. Consequently, there is no clear biological indicator for burnout that is sensitive and specific enough to confirm the diagnosis of burnout. In addition, the majority of studies focused on measurements of circulating proteins (mainly cortisol), but lack an in-depth understanding of underlying molecular mechanisms affecting their expression^8^.

A potential role of the Brain Derived Neurotrophic Factor (BDNF) in burnout has recently been suggested^9^. BDNF is a neurotrophic protein that plays a critical role in the development and maintenance of normal brain function, due to its importance in learning and memory^10^ and its key regulating function in neuronal differentiation, and neurite and synaptic growth^11^. Impaired levels of BDNF have been widely studied in the context of stress-related psychiatric outcomes like major depression and anxiety disorders, both in animal models and human studies^12–14^. Interestingly, in two recent studies, decreased BDNF serum levels were found in people with burnout, suggesting a potential role of BDNF in the neurobiology of this phenomenon^15,16^. Despite clear indications for changes in BDNF protein expression in stress-related outcomes, the exact biological mechanisms driving these changes are largely unknown.

BDNF protein expression is regulated through epigenetic mechanisms occurring in the *BDNF* gene. This gene is located on chromosome 11 and has a complex structure, consisting of 11 exons (I–IX, Vh and VIIIh), nine of them having a functional promoter (exons I–VII and IX)^17^. Different *BDNF* transcripts can be generated using combinations of alternative promoters and splicing mechanisms, which determines a tissue-specific BDNF expression regulation^18^. In the context of stress-related mental disorders, DNA methylation changes of CpG islands overlapping with promoters of exon I and exon IV of the *BDNF* gene have been most thoroughly studied^19,20^. In addition, methylation of exon IX gained attention in research as this region contains a common single-nucleotide polymorphism (SNP) (rs6265), which causes a valine (Val) to methionine (Met) substitution at codon 66. This SNP is thought to alter *BDNF* methylation levels and has been widely implicated in vulnerability to mental health disorders^18,21,22^.

Despite existing evidence of the potential role of BDNF in biological processes occurring in burnout, to the best of our knowledge, there are no studies investigating potential underlying epigenetic (DNA methylation) mechanisms. DNA methylation changes are sensitive to the environment, stable and reversible, and can therefore provide a significant contribution to our knowledge about the disruption of specific pathways occurring in burnout. Therefore, our aim is to comprehensively investigate the role of BDNF in burnout, by focusing on Val66Met polymorphism, DNA methylation of several gene regions (promoter I and IV and exon IX), and sBDNF protein levels.

## Methods

### Study population

**Fig. 1:**
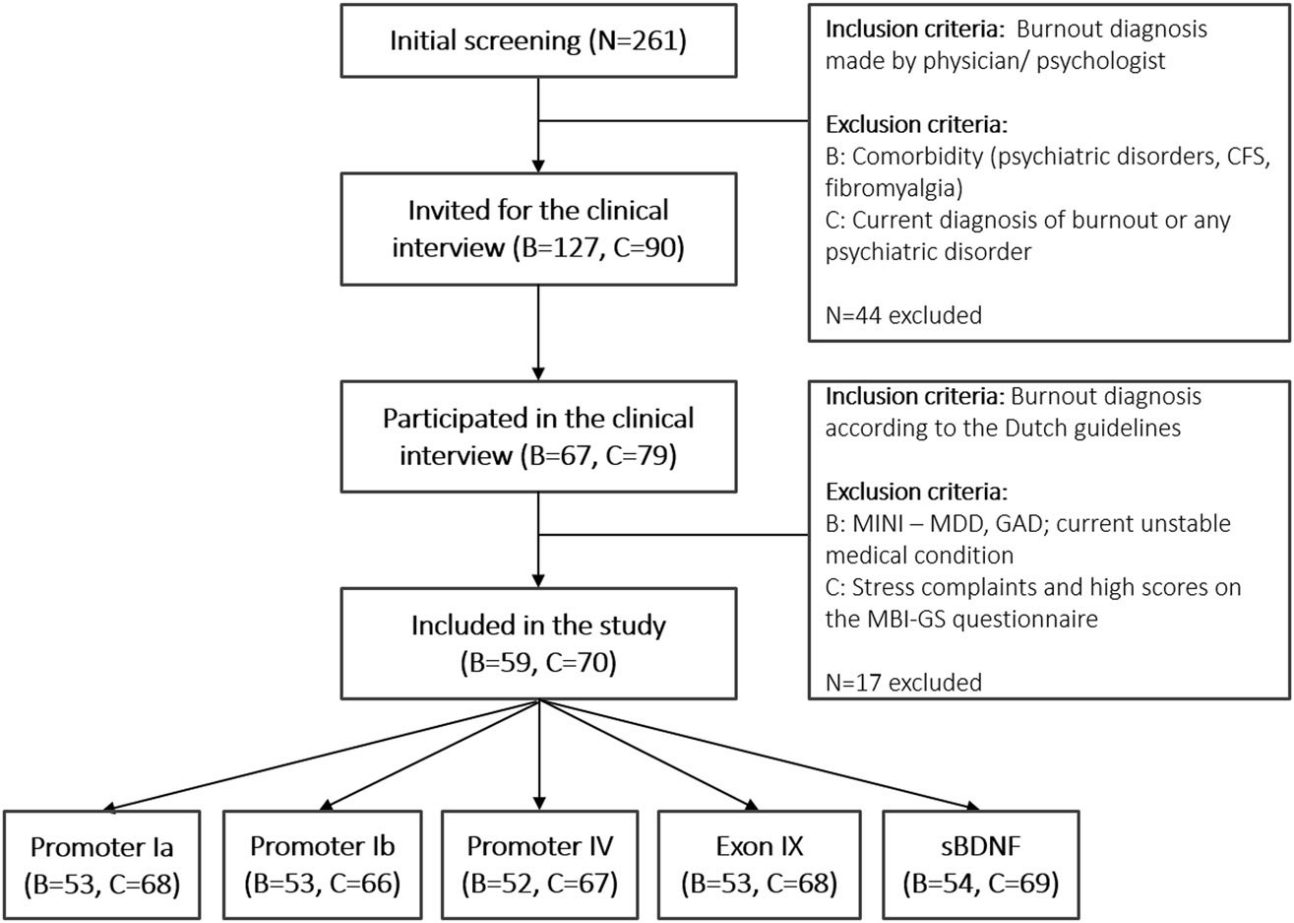
Overview of the inclusion process. B: burnout group; C: control group; CFS: chronic fatigue syndrome; MINI: Mini-International Neuropsychiatric Interview; MDD: major depressive disorder; GAD: general anxiety disorder; MBI-GS: Maslach Burnout Inventory – General Survey; sBDNF: serum BDNF protein expression.

We conducted a cross-sectional study by recruiting individuals with burnout and healthy controls. Recruitment of both burnout subjects and healthy controls was performed in two steps. First, information about the study was disseminated using media channels, communication with organizations specialised in burnout prevention and treatment, and flyers distributed at KU Leuven and UZ Leuven facilities. All interested potential participants were asked to fill in an online screening tool including questions about the burnout diagnosis and comorbidity. People who reported a diagnosis of burnout made by a physician or psychologist and without comorbidity were considered for inclusion in the burnout group. Among potential candidates for the control group, people who reported current diagnosis of burnout or any psychiatric disorder were excluded. Subject who satisfied the inclusion/exclusion criteria in the initial assessment were invited for a clinical interview with a psychologist. During this interview, burnout was assessed based on the Dutch practice guidelines for managing adjustment disorders in occupational and primary health care^23^. In addition, all participants were screened for major depressive disorder and general anxiety disorder, by using MINI^24^ and those fulfilling criteria were excluded. Finally, we included 129 individuals in the study (*N* = 59 for the burnout group and *N* = 70 for the control group). All subjects were of Caucasian origin. An overview of the inclusion process is given in Fig. 1.

This study was approved by the commission for Medical Ethics of the UZ Leuven (S59567) and all subjects gave their informed consent priory to inclusion in the study.

### Psychological measures

A clinical interview was used to confirm the burnout diagnosis, assess inclusion and exclusion criteria and record data on factors related to burnout (e.g., medication use). In order to measure burnout symptoms dimensionally, all participants were asked to fill in a validated Dutch version of the Maslach Burnout Inventory—General Survey (MBI-GS)^25^, which is the Utrecht Burnout Scale-A (UBOS-A)^26^. According to MBI-GS, burnout is assessed by three dimensions: exhaustion, cynicism and reduced professional efficacy. People from the control group who reported stress complaints during the interview and scored high on the UBOS-A questionnaire (exhaustion > 21 and cynicism > 14) were excluded from further analyses. In order to measure depressive symptoms, the Dutch version of the Beck Depression Inventory II (BDI-II) was used^27^. We further divided all items in the BDI-II questionnaires into cognitive-affective and somatic-vegetative depressive symptoms, according to Bridwell^28^. Finally, questions were included about smoking habits and alcohol consumption, as well as socio-demographic characteristics, such as age, gender, education, and work-related information (e.g., sector, sickness absence etc.).

### Sample collection and DNA extraction

Venous blood was drawn from participants and collected in two ethylenediaminetetraacetic acid (EDTA) tubes, and in one tube with clot activator (BD Vacutainer®). One EDTA tube was immediately processed for determination of differential blood cell count, whereas the other was stored at −80 °C for DNA methylation analysis. Tubes with clot activator were left to coagulate for 30 min at room temperature and centrifuged at 2000 × *g* for 10 min. Serum aliquots were separated after centrifugation and stored at −80 °C until ELISA assay was performed.

All samples were randomised prior to DNA extraction. DNA extraction from whole blood was performed, using the QIAamp DNA Blood Mini Kit (Qiagen Inc., Valencia, CA). The final elution volume obtained was 100 μL. The quantity and purity of DNA were determined by a NanoDrop spectrophotometer.

### DNA methylation and Val66Met polymorphism analysis

Both epigenetic (DNA methylation) and genetic (polymorphism) analysis were done using pyrosequencing. Genomic DNA extracted from whole blood was bisulfite-converted using the EZ-96 DNA Methylation-Gold™ Kit (#D5008, Zymo Research). Pyrosequencing was performed using Pyro Gold reagents (#970802, Qiagen) on the PyroMark Q24 instrument (Qiagen) following the manufacturer’s instructions. Pyrosequencing results were analysed using the PyroMark analysis 2.0.7 software (Qiagen). The analysed sequences include 8 CpGs in the promoter of exon I (4 in promoter Ia and 4 in promoter Ib), 7 CpGs in promoter of exon IV and 5 CpGs in a coding region of exon IX (including Val66Met polymorphism) of the *BDNF* gene. All samples were run in singlets as the reproducibility test on three random samples showed low variability for all four assays (SD < 1%). Positive control DNA (highly methylated) was used for both assay validation and validation of each pyrosequencing analysis. A detailed protocol with all analysed amplicons, PCR and sequencing primers, and reproducibility test is provided in Supplementary information 1.

### BDNF ELISA measurements

BDNF concentration in randomised serum samples of all participants was measured by sandwich enzyme linked immunosorbent assay (ELISA) using a commercially available Human BDNF ELISA Kit (Biotrend, Cologne, Germany), according to the manufacturer’s instructions. The BDNF concentrations of samples were calculated according to the standard curve for each plate.

### Statistical analysis

First, we compared socio-demographic, clinical variables and genotype of the burnout and the control group using an independent sample *t*-test for continuous variables, and Chi-Square test for categorical variables.

To avoid multiple testing, we applied a linear mixed model in all analyses testing associations with DNA methylation. In these models, DNA methylation was used as a response variable. Other variables for which association with DNA methylation was tested were used as explanatory variables (group—burnout/control, burnout or depressive symptoms, polymorphism or sBDNF) in separate models. CpG site was always included as an explanatory variable, as well as the interaction between CpG site and the other explanatory variable of interest. A significant interaction test implies that the association between the variable of interest and CpG methylation is different between the individual CpGs. If this was the case, results were reported per individual CpG. In the case of a non-significant interaction test, the main effect of the variable of interest over the different CpGs was reported. This approach was used to test the group differences in methylation (burnout vs. control), associations between burnout and depressive symptoms (continuous variables) and DNA methylation, Val66Met polymorphism and DNA methylation and sBDNF levels and DNA methylation. A random effect (subject) was modelled to deal with the clustered nature of the data. If appropriate, other covariates were included in the model to correct for possible confounding and included age, gender, current smoking (yes/no), antidepressant use (yes/no), and white blood cell count.

Moreover, we used an independent sample *t*-test to compare sBDNF levels between the two groups, since the BDNF concentrations were normally distributed among all subjects (Kolmogorov–Smirnov one-sample test: *p* > 0.05). Linear regression analysis was used to assess the impact of burnout symptoms (exhaustion, cynicism and professional efficacy), and depressive symptoms on BDNF protein levels as the outcome variable.

All statistical analyses have been performed using SPSS software package, version 25.0. All tests were two-sided, and the significance level was set at 0.05.

## Results

### Study population

Subjects with burnout and healthy controls did not differ significantly in sex and habitual smoking. However, mean age was significantly higher in the burnout group (48.5 ± 8) compared to the control group (38.2 ± 12.1). Moreover, 42.4% of subjects with burnout reported taking antidepressants, as opposed to 2.8% of participants in the control group (see Table 1). Regarding psychological assessment using self-reported tools, burnout subjects scored significantly higher on the exhaustion and cynicism scales of MBI-GS and significantly lower on the professional efficacy. In addition, they reported significantly higher symptoms of depression (BDI-II). Interestingly, participants with burnout reported more somatic-vegetative depressive symptoms (9.3 ± 3.8) compared to cognitive-affective (5.8 ± 4.4).

**Table 1:** Socio-demographic and clinical characteristics of participants.

	Control group	Burnout group	Significance
*N*	70	59	
**Age (year)**	38.2 ± 12.1	48.5 ± 8	*t* = −5.53, df = 125 *p* < 0.001**
Proportion of women (%)	64.3**%**	64.4**%**	X^2^ < 0.001 *p* = 0.989
**Education (%)**			
Primary	0%	1.7%	X^2^ = 10.6
Lower secondary	4.3%	5.1%	*p* = 0.032*
Higher secondary	8.6%	16.9%	
Non-university higher	37.1%	52.6%	
University degree	50%	23.7%	
**Habitual smoking (%)**			
Yes	10%	8.5%	X^2^ = 0.6
No	90%	91.5%	*p* = 0.430
**Antidepressant use**			
Yes	2.8%	42.4%	X^2^ = 30.2
No	97.2%	57.6%	*p* < 0.001**
**Currently on sick leave (%)**			
Yes	0%	63%	X^2^ = 61.9
No	100%	37%	*p* < 0.001**
**Psychological assessment**			
Exhaustion (MBI-GS)	13.6 ± 4.6	28.4 ± 6.9	*p* < 0.001**
Cynicism (MBI-GS)	8.9 ± 3.7	18 ± 6.2	*p* < 0.001**
Professional efficacy (MBI-GS)	32.6 ± 5.4	28 ± 7	*p* < 0.001**
Depressive symptoms (BDI-II)	5.1 ± 5.8	20.9 ± 9.6	*p* < 0.001**
• Cognitive-affective	1.2 ± 2.1	5.8 ± 4.4	*p* < 0.001**
• Somatic-vegetative	2.3 ± 2.5	9.3 ± 3.8	*p* < 0.001**
**Genotype frequency (%)**			
GG	66.2%	60.4%	X^2^ = 0.43
AG	27.9%	37.7%	*p* = 0.511
AA	5.9%	1.9%	

Age and data from self-reported questionnaires (psychological assessment) are displayed as mean ± standard deviation. *p*-Values are derived from statistical analysis using independent sample *t*-test for continuous variables or Chi-Square test for categorical variables. *MBI-GS* Maslach Burnout Inventory—General Survey, *BDI-II* Beck Depression Inventory II. Significance: *p < 0.05, **p < 0.01.

### Val66Met polymorphism

In the overall sample, the frequency of Val homozygotes (G/G) was 63.7%, whereas 32.2% of participants were heterozygote Met carriers (G/A) and 4.1% were Met homozygotes (A/A). These percentages are in line with previous analysis of Val66Met frequencies in Caucasians^29^ and Belgian population^30^. As previously suggested, we grouped the Met carriers together (G/A and A/A). Similar distribution was observed in each group separately. In the burnout group, 32 participants were Val carriers (60.4%) and 21 participants (39.6%) were Met carriers (20A/G and 1A/A). Among healthy participants, 45 were Val carriers (66.2%) as opposed to 23 (33.8%) Met carriers (19A/G and 4A/A). The number of Met carriers did not differ significantly between the groups (*X*^2^ = 0.43, *p* = 0.511), and the polymorphism did not have an effect on self-reported symptoms of burnout (data not shown). The Val66Met polymorphism was associated with DNA methylation of the surrounding CpG site in exon IX in such way that Met carriers had decreased methylation levels (mean difference 42.59%, *p* < 0.001). In addition, Met carriers had increased methylation in promoter Ia (mean difference 0.31%, *p* = 0.029) whereas no effect was observed on the methylation levels in other regions (all *p*-values > 0.05). sBDNF concentrations did not differ between Val and Met carriers (*t* = 0.18, *p* = 0.857).

### Association between burnout and DNA methylation

**Fig. 2:**
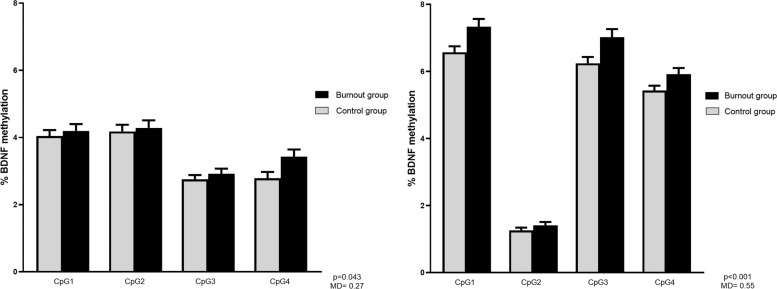
Overview of *BDNF* methylation in promoter of exon I (region Ia and Ib). Mean methylation values and standard error of the mean in the control and the burnout group for region Ia (**a**) and Ib (**b**) of *BDNF* promoter I. Since no interaction between the group (control vs burnout) and the CpG was observed in the mixed model analysis, differences in individual CpGs were not compared. Instead, the presented values for the mean methylation difference between groups and the *p*-value reflect a cumulative effect, across all CpGs.

We found significantly increased methylation in the burnout group for promoter Ia (mean difference 0.27%, *p* = 0.043) and promoter Ib (mean difference 0.55%, *p* < 0.001) and this was an overall effect in the whole region (not CpG-specific). When confounders were added to the model (age, sex, smoking, antidepressant use and white blood cell count), the group difference remained significant only for promoter Ib (mean difference 0.42%, *p* = 0.005). An overview of *BDNF* methylation of promoter I (region Ia and Ib) is displayed in Fig. 2. In addition, we found a significant association between DNA methylation in promoter Ib and all burnout dimensions: exhaustion (B = 0.14, *p* < 0.001), cynicism (B = 0.15, *p* < 0.001), and professional efficacy (B = −0.12, *p* = 0.034), as well as depressive symptoms (B = 0.02, *p* < 0.001) (for a detailed overview, see Supplementary information 2).

**Fig. 3:**
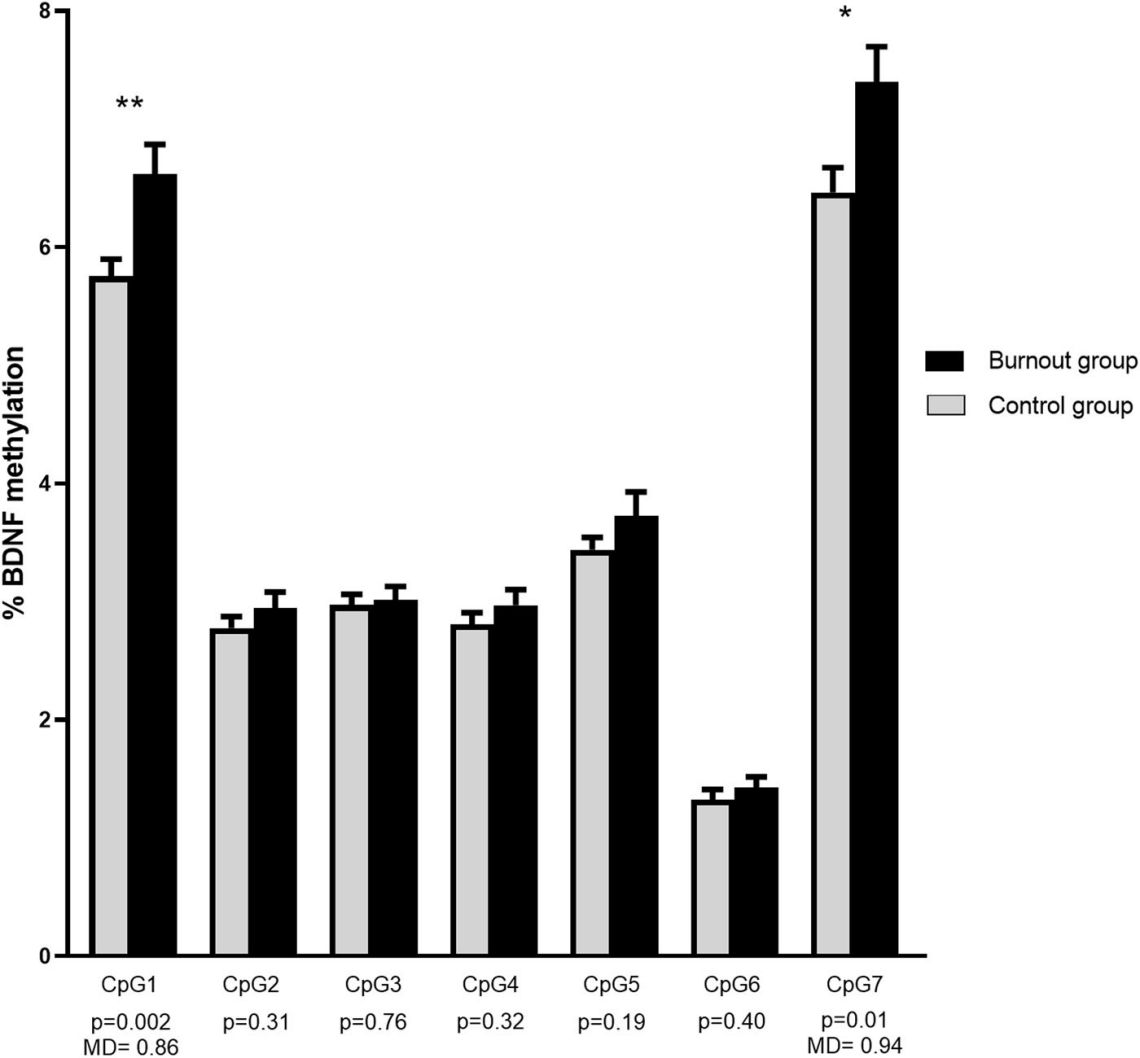
Overview of *BDNF* methylation in promoter of exon IV. Mean methylation values and standard error of the mean in the control and the burnout group for *BDNF* promoter IV. Since an interaction between the group (control vs burnout) and the CpG was observed in the mixed model analysis, the mean methylation difference between groups and the *p*-value are presented for each CpG separately. Significance: *p < 0.05, **p < 0.01.

Similarly, we found increased DNA methylation of *BDNF* promoter IV in the burnout group (mean difference 0.36%, *p* < 0.001). In addition, this effect was CpG-specific and post hoc analysis revealed that the methylation difference lies on CpG1 (mean difference 0.86%, *p* = 0.002) and CpG7 (mean difference 0.94%, *p* = 0.01). This association remained significant when all covariates were added to the model for both CpG1 (mean difference 0.81%, *p* = 0.024) and CpG7 (mean difference 1.07%, *p* = 0.02). An overview of *BDNF* methylation in promoter IV is displayed in Fig. 3. Moreover, CpG1 methylation was significantly correlated with self-reported symptoms of burnout: exhaustion (B = 0.16, *p* = 0.032) and cynicism (B = 0.23, *p* = 0.004), but not with professional efficacy (B = −0.20, *p* = 0.14). In addition, CpG1 methylation was significantly associated with symptoms of depression (B = 0.03, *p* = 0.03). Similar results were observed for CpG7. DNA methylation of this CpG was significantly associated with symptoms of exhaustion (B = 0.26, *p* = 0.008), cynicism (B = 0.22, *p* = 0.038) and professional efficacy (B = −0.53, *p* = 0.001), as well as depressive symptoms (B = 0.04, *p* = 0.02).

Finally, we neither found a significant difference in DNA methylation of *BDNF* exon IX between the groups nor an association between burnout and depressive symptoms and DNA methylation of this region.

### Association between *BDNF* DNA methylation and sBDNF levels

Next, we investigated the correlations between DNA methylation of the *BDNF* gene (promoter I, IV and exon IX) and sBDNF levels. We found a significant negative correlation between DNA methylation of promoter I and sBDNF, for both promoter Ia (B = −0.01, *p* = 0.029) and promoter Ib (B = −0.03, *p* < 0.001), without an interaction effect with the CpG site (*p* = 0.36 for promoter Ia; *p* = 0.54 for promoter Ib). When all confounders were added to the model (age, sex, smoking, antidepressant use, white blood cell count), this correlation remained significant for both promoter Ia (B = −0.01, *p* = 0.029) and Ib (B = −0.01, *p* = 0.011). For promoter IV and exon IX, we observed a negative but nonsignificant association between DNA methylation and the sBDNF levels (promoter IV: B = −0.01, *p* = 0.092; exon IX: B = −0.03, *p* = 0.459). Finally, polymorphism did not have a moderation effect on any of the associations between DNA methylation and sBDNF levels (all *p-*values > 0.05).

### Association between burnout and sBDNF levels

**Fig. 4:**
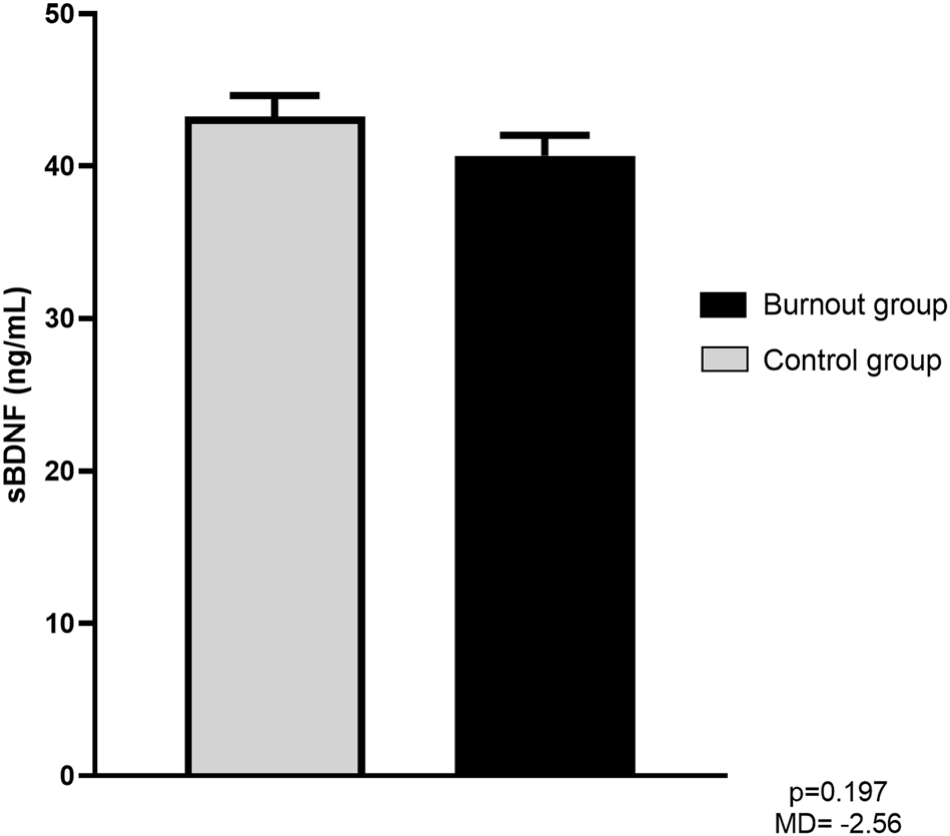
Overview of sBDNF levels. Mean sBDNF concentration and standard error of the mean in the control and the burnout group. Comparison between the groups was analysed using an independent sample *t*-test. The mean difference in the concentration and the *p*-value are also presented.

Regarding the sBDNF levels, we did not observe statistically significant differences between the levels in the burnout group compared to the healthy control group (mean difference 2.56 ng/mL, SE = 1.97, *p* = 0.197). When controlled for confounders (age, sex, smoking, antidepressant use and white blood cell count), the observed effect remained nonsignificant (*p* = 0.145). However, we also observed low power in this analysis (0.307). A comparison of sBDNF levels between the groups is depicted in Fig. 4.

In addition, linear regression analysis showed that there was no significant effect of burnout symptoms on sBDNF (exhaustion: B = −0.77, *p* = 0.149; cynicism: B = −0.84, *p* = 0.144; professional efficacy: B = 0.61, *p* = 0.503). Interestingly, we observed a significant negative correlation between the presence of depressive symptoms and sBDNF levels (B = −0.26, *p* = 0.004). In addition, when we performed separate analyses with cognitive-affective and somatic depressive symptoms in the burnout group only, it seemed that this correlation is more driven by cognitive symptoms than by somatic symptoms. In other words, cognitive-affective symptoms were more strongly correlated with the sBDNF levels (R^2^ = 0.067, B = −0.71, *p* = 0.004) compared to somatic symptoms (R^2^ = 0.037, B = −0.44, *p* = 0.034). Finally, higher sBDNF concentrations were associated with a higher number of white blood cells in our sample (B = 1.294, *p* = 0.018).

## Discussion

In the present study we investigated the potential role of BDNF in burnout, by focusing on DNA methylation of specific regions (promoter I, IV and exon IX) and sBDNF protein levels. We found increased methylation of promoter I and IV in the burnout subjects, compared to healthy controls, which also positively correlated with burnout symptoms. Moreover, we observed a negative correlation between methylation of promoter I and sBDNF levels, confirming a silencing effect of DNA methylation in the promoter region. This is in line with the previous literature^31–34^. Importantly, the observed associations remained significant when we controlled for differential white blood cell count, which is pointed out as one of the major potential confounders for DNA methylation analysis using peripheral blood samples^35,36^. Finally, we did not observe significant differences in sBDNF protein levels between the groups.

To our knowledge, this is the first study to observe changes in DNA methylation of the *BDNF* gene in a burnout sample and therefore these findings are of added value for understanding epigenetic mechanisms occurring in burnout. Interestingly, Song et al.^37^. reported increased methylation levels of the whole *BDNF* gene in workers exposed to high work stress. Moreover, in several other studies increased DNA methylation levels of *BDNF* promoter I and IV were observed in patients with major depression^38–40^, bipolar disorder^41^, borderline personality disorder^42^ and schizophrenia^43,44^. Therefore, these changes seem to refer to potential shared underlying mechanisms for the development of a wide spectrum of stress-related phenotypes.

Another novel finding in our study is the negative correlation between DNA methylation of *BDNF* promoter I and sBDNF levels. To the best of our knowledge, this is the first study investigating this link in a humans. The effect of methylation changes in promoter I on BDNF expression have been previously demonstrated in animal models^45–47^. Even though the exact mechanisms of *BDNF* gene silencing caused by promoter hypermethylation are only partially known, it is suggested that DNA-binding proteins, such as MeCP2, recruit chromatin silencing complexes that further act via other epigenetic mechanisms, such as histone methylation or deacetylation. Overall, our findings together with those from previously published studies suggest that *BDNF* gene silencing is mediated by hypermethylation in the promoter I region of the gene.

In our study we did not find evidence for a link between DNA methylation of promoter IV and sBDNF protein levels. The reason could be that we looked only at one part of the CpG island^13^. Therefore, the observed changes might not have an impact on BDNF expression on its own but rather contribute to a cumulative effect of changes in a larger number of CpGs, as we observed in promoter I. Another explanation could be that we looked at the total BDNF protein expression at the peripheral level, rather than its specific isoforms. In a recent study^13^, the authors compared expression of BDNF isoforms in peripheral leucocytes and hippocampus and showed that the BDNF IV isoform is comparable in these two tissues, whereas BDNF isoform I was more highly expressed in leucocytes. Even though the BDNF protein passes the blood-brain barrier^48^, at least part of the BDNF measured in serum most likely originates from leucocytes^49^ and platelets^50^. Consequently, BDNF protein levels in serum could be more driven by changes in promoter of exon I, whereas changes in promoter of exon IV could be more visible in the brain tissue. This is also supported by the direct correlation between white blood cell count and sBDNF, as observed in our study.

Interestingly and somewhat surprisingly, we did not observe a significant difference in sBDNF between individuals with burnout and healthy controls. This is in contrast to the two previously published studies where significantly decreased sBDNF levels were linked to burnout^15,16^. The reason why we were not able to detect differences in sBDNF could be the fact that we collected the serum samples at different hours throughout the day. Consequently, a higher variability of our samples and a lower power to detect significant differences might be the reason why we obtained such results. Nevertheless, we did observe a negative correlation between depressive symptoms and sBDNF levels, which was statistically significant. Interestingly, sBDNF levels were more strongly correlated with cognitive-affective symptoms than somatic-vegetative, which might indicate that people with more severe burnout, who are at higher risk of developing major depression have more pronounced epigenetic changes, which result in changes in BDNF expression visible at the peripheral level. Nevertheless, our conclusions are limited due to the cross-sectional design and need to be supported by longitudinal data.

Changes in BDNF protein level induced by chronic stress are generally thought to contribute to the development of symptoms via their effect on hippocampal neurogenesis^51^. However, the exact mediating mechanisms are still unclear. Eriksson and Wallin suggested that decreased hippocampal neurogenesis induced by changes in HPA-axis reactivity and excessive cortisol exposure plays a major role in stress-related syndromes like burnout^52^. At a molecular level, it is generally accepted that effects of chronically elevated cortisol levels are achieved via modulations of glucocorticoid receptors in the hippocampus, which can modulate gene expression and induce DNA methylation changes^53^. Moreover, a recent study showed that elevated cortisol modulated mRNA expression of the total BDNF as well as isoforms IV, VI and IX in an in vitro model of human hippocampal cells^54^. However, DNA methylation changes were not investigated. Therefore, additional in vitro experiments could contribute to a further understanding of the potential role of DNA methylation as a mediator of changes in the BDNF expression and complement findings of clinical studies.

Finally, we did not find evidence for the role of genotype in BDNF-related mechanisms present in burnout. In our study, we did not observe differences in Val66Met polymorphism between the two groups. Moreover, Val66Met polymorphism did not have an impact on sBDNF levels nor did it moderate the observed associations between DNA methylation and sBDNF protein expression. Even though there is some evidence in the literature on the role of genetic factors in burnout^55^, we found no studies focusing on Val66Met specifically. Despite that the presence of a Met allele is considered as a risk factor for vulnerability to mental disorders^56^, these findings are inconsistent and were mainly related to more severe mental disorders such as major depressive disorder, schizophrenia and posttraumatic stress disorder^21^. In contrast, our findings suggest that environment-induced epigenetic changes in burnout seem to play a more important role than genetic factors in the context of biological mechanisms involving BDNF.

## Conclusions

In conclusion, this is the first study to provide evidence that DNA methylation changes in the *BDNF* gene promoters might play an important role in the downregulation of the BDNF protein levels in burnout. Moreover, although we noted a potential effect of burnout severity and presence of depressive symptoms on the observed changes, additional longitudinal studies are needed to understand the exact dynamics and potential reversibility of these changes throughout different clinical stages and recovery.

## Supplementary information

Supplementary information 1.

Supplementary information 2.
