# Supplementary material for: Epigenetic perspective on the role of brain-derived neurotrophic factor in burnout
Source: Transl Psychiatry. 2020 Oct 19;10:354. doi: 10.1038/s41398-020-01037-4 (PMC7573604; doi:10.1038/s41398-020-01037-4)
Supplement: Supplementary file 1 — Supplementary information 1. [file 41398_2020_1037_MOESM1_ESM.docx]

**Supplementary information 1**

Assay validation and pyrosequencing protocol

200 ng of genomic DNA was bisulfite converted using the EZ-96 DNA Methylation-Gold™ Kit (#D5008, Zymo Research). Converted DNA was eluted with 35 μL of M-elution buffer. Next, 1 μL of converted DNA was amplified by PCR in a total volume of 25 μL containing 0.2 μM of primers and 2× Qiagen PyroMark PCR Master Mix (#978703, Qiagen). All analysed amplicons are given in **Supplementary table 1.** The analyzed sequences include two regions that lie within promoter of exon I (promoter Ia and promoter Ib), one region within promoter of exon IV and a coding region in exon IX of the *BDNF* gene. Primers overlapping with promoter of exon I were ordered from Qiagen (#PM00155540 and #PM00155547 PyroMark CpG Assays). Primer sequences for promoter IV and exon IX were designed using PyroMark Assay Design SW 2.0 (Qiagen). The sequence of exon IX included Val66Met polymorphism (rs 6265). **Supplementary table 2.** provides an overview of PCR and sequencing primers.

The assays were validated according to the validation steps recommended in the pyrosequencing manual (PyroMark Q24 User Manual, Qiagen). This included testing the following controls: 1) PCR without template DNA, 2) PCR with template DNA but no sequencing primer, 3) Sequencing primer without any PCR product, 4) Biotinylated primer without any PCR product, 5) Sequencing primer and biotinylated primer together without PCR product. These tests were run for each of the four assays and they all resulted in absence of signal.

PCR amplification was performed according to the cycling conditions presented in **Supplementary table 3.** As previously reported (1), during optimization of the PCR protocol, we performed gel electrophoresis for the PCR products amplified with annealing temperatures in the range 50-60 °C to determine the optimal cycling conditions, which were then further applied. The expected product sizes of the PCR amplicons were 182 (promoter Ia), 118 bp (promoter Ib), 321 bp (promoter IV) and 398 bp (exon IX).

Subsequently, 20 μL of biotinylated PCR product was immobilized to Streptavidin Sepharose High Performance beads (#17-5113-01, GE Healthcare) followed by annealing to 25 μL of 0.3 μM sequencing primer at 80 °C for 2 min followed by a 10 min cooling down period. Pyrosequencing was performed using Pyro Gold reagents (#970802, Qiagen) on the PyroMark Q24 instrument (Qiagen) following the manufacturer’s instructions. Pyrosequencing results were analysed using the PyroMark analysis 2.0.7 software (Qiagen). Eight CpGs were analysed for promoter of exon I (four for promoter Ia and four for promoter Ib), seven CpGs promoter of exon IV, and five CpGs for of exon IX. 100% methylated control DNA (Qiagen) was applied with every sample batch for technical variation analysis.

**Supplementary table 1.** Overview of analyzed amplicons

The sequence of exon IX includes Val66Met polymorphism (rs 6265), which is marked G/A in the corresponding sequence. All CG pairs (CpGs) assessed for methylation are marked in bold.

| **Region** | **Strand** | **Analyzed amplicon** |
| --- | --- | --- |
| Promoter of exon Ia | Sense | CTG CAT G**CG** T**CG** AAG **CGC G**A |
| Promoter of exon Ib | Antisense | TTA CTT TC**C** **G**CC AAC A**CG** TGA CCT CTT **CG**C TTC CCA GCT TG**C G**T |
| Promoter of exon IV | Sense | TTA T**CG** **CG**G AGA GGG TTG TTT T**CG** TTG T**CG** TTT TTT T**CG** G**CG** AAT TAG TAT GAA ATT TTT TTG TTT TTG T**CG** AGA TTA AAT GGA GTT TTT |
| Coding region of exon IX | Antisense | TAT TAT TGG TTG ATA TTT T**CG** AAT A**CG/A** TGA TAG AAG AGT TGT TGG ATG AGG ATT AGA AAG TT**C** **G**GT TTA ATG AAG AAA ATA ATA AGG A**CG** TAG ATT TGT ATA **CG**T TTA GGG TGA TGT TTA GTA GTT AA |

**Supplementary table 2.** Overview of PCR and sequencing primers for DNA methylation analysis

To assess DNA methylation in promoter of exon I, we used commercially available assays designed by QIAGEN and we report the product codes and analysed sequences. For DNA methylation of promoter of exon IV and the coding region of exon IX, we designed PCR and sequencing primers using PyroMark Assay Design SW 2.0 (Qiagen).

| **Region** | **PCR primer** | **Pyrosequencing primer** | **Number of CpGs** |
| --- | --- | --- | --- |
| Promoter of exon Ia | P8_PM00155540 | P8_PM00155540 | 4 |
| Promoter of exon Ib | P9_PM00155547 | P9_PM00155547 | 4 |
| Promoter of exon IV | Forward: GGG TTG GAA GTG AAA ATA TTT GTA AA  Reverse: /5Biosg/CC CCA TCA ACC AAA AAC TCC ATT TAA TC | GTG GAT TTT TAT TTA TTT TTT TAT | 7 |
| Coding region of exon IX | Forward: ATG AAG GTT GTT TTT ATG AAA GAA GTA  Reverse: /5Biosg/AC CCA CTC ACT AAT ACT A | AGG TTT AAG AGG TTT GA | 5 |

**Supplementary table 3.** Overview of PCR cycling conditions

| **Region** | **Cycling conditions** |
| --- | --- |
| Promoter of exon Ia | 95 °C 15’00’’  45x (94 °C 30’’, 56 °C 30’’, 72 °C 30’’)  72 °C 10’00’’  4°C ∞ |
| Promoter of exon Ib | 95°C 15’00’’  45x (94 °C 30’’, 56 °C 30’’, 72 °C 30’’)  72 °C 10’00’’  4°C ∞ |
| Promoter of exon IV | 95 °C 15’00’’  45x (94 °C 30’’, 54 °C 30’’, 72 °C 30’’)  72 °C 10’00’’  4°C ∞ |
| Coding region of exon IX | 95 °C 15’00’’  45x (94 °C 30’’, 54 °C 30’’, 72 °C 30’’)  72 °C 10’00’’  4 °C ∞ |

**Supplementary table 4.** Overview of reproducibility analysis performed on three random samples and a positive control for each assay, which were run in triplicates. DNA methylation (DNAm) was presented as the average % and variability was expressed as standard deviation (SD) of the three replicates.

| **Name** | **Average DNAm %**  **Replicate 1** | **Average DNAm %**  **Replicate 2** | **Average DNAm %**  **Replicate 3** | **Variability (SD)** |
| --- | --- | --- | --- | --- |
| **Promoter of exon Ia**  **Sample 1** | 3.9475 | 5.235 | 4.0275 | 0.72 |
| **Promoter of exon Ia**  **Sample 2** | 3.0975 | 4.7175 | 4.2525 | 0.83 |
| **Promoter of exon Ia**  **Sample 3** | 3.4625 | 3.6975 | 3.5225 | 0.12 |
| **Promoter of exon Ia**  **Positive control** | 87.9925 | 79.5075 | 82.0275 | 4.36 |
| **Promoter of exon Ib**  **Sample 1** | 5.8525 | 6.155 | 6.515 | 0.33 |
| **Promoter of exon Ib**  **Sample 2** | 5.135 | 5.595 | 5.65 | 0.28 |
| **Promoter of exon Ib**  **Sample 3** | 4.2775 | 4.225 | 3.805 | 0.26 |
| **Promoter of exon Ib**  **Positive control** | 85.1225 | 91.625 | 87.3525 | 3.30 |
| **Promoter of exon IV**  **Sample 1** | 3.3314 | 3.1986 | 3.4029 | 0.10 |
| **Promoter of exon IV**  **Sample 2** | 3.0414 | 3.03 | 3.1271 | 0.12 |
| **Promoter of exon IV**  **Sample 3** | 4.7543 | 4.4514 | 5.02 | 0.29 |
| **Promoter of exon IV**  **Positive control** | 90.3771 | 90.0529 | 90.9629 | 0.46 |
| **Exon IX**  **Sample 1** | 78.912 | 80.254 | 78.656 | 0.86 |
| **Exon IX**  **Sample 2** | 85.418 | 84.05 | 84.424 | 0.71 |
| **Exon IX**  **Sample 3** | 86.202 | 86.324 | 85.386 | 0.51 |
| **Exon IX**  **Positive control** | 69.21 | 70.62 | 72.292 | 1.54 |
