# Supplementary material for: Epigenetic perspective on the role of brain-derived neurotrophic factor in burnout
Source: Transl Psychiatry. 2020 Oct 19;10:354. doi: 10.1038/s41398-020-01037-4 (PMC7573604; doi:10.1038/s41398-020-01037-4)
Supplement: Supplementary file 2 — Supplementary information 2. [file 41398_2020_1037_MOESM2_ESM.docx]

**Supplementary information 2**

**Supplementary table 1.** Between group differences in DNA methylation and serum BDNF (sBDNF) protein levels

Data are expressed as mean (SD). Group differences were analyses using a mixed model for DNA methylation and independent sample *t*-test for sBDNF.

|  | Control group | Burnout group | Group differences | | |
| --- | --- | --- | --- | --- | --- |
|  | Mean (SD) | Mean (SD) | Mean difference | 95% CI | *p*-value |
| DNA methylaition (%) |  |  |  |  |  |
| Promoter exon Ia  All CpGs^a^ | 3.44 (1.58) | 3.71 (1.55) | 0.27 | 0.01, 0.53 | **0.043** |
| Promoter exon Ib  All CpGs^a^ | 4.87 (2.47) | 5.42 (2.77) | 0.55 | 0.3, 0.79 | **<0.001** |
| Promoter exon IV  CpG1^b^ | 5.75 (1.18) | 6.61 (1.84) | 0.86 | 0.31, 1.41 | **0.002** |
| Promoter exon IV  CpG2^b^ | 2.78 (0.80) | 2.94 (0.98) | 0.17 | -0.16, 047 | 0.308 |
| Promoter exon IV  CpG3^b^ | 2.97 (0.75) | 3.01 (0.83) | 0.04 | -0.24, 0.33 | 0.764 |
| Promoter exon IV  CpG4^b^ | 2.81 (0.80) | 2.97 (0.95) | 0.16 | -0.16, 0.48 | 0.324 |
| Promoter exon IV  CpG5^b^ | 3.44 (0.87) | 3.72 (1.48) | 0.29 | -0.14, 0.72 | 0.192 |
| Promoter exon IV  CpG6^b^ | 1.32 (0.70) | 1.43 (0.64) | 0.10 | -0.14, 0.35 | 0.403 |
| Promoter exon IV  CpG7^b^ | 6.46 (1.73) | 7.40 (2.15) | 0.94 | 0.23, 1.64 | **0.01** |
| Coding region of exon IX  All CpGs^a^ | 80.0 (16.11) | 80.74 (14.95) | 0.74 | -0.92, 2.4 | 0.384 |
| sBDNF protein expression (ng/mL) | 43.28 (11.59) | 40.69 (9.83) | -2.56 | -6.46, 1.34 | 0.197 |

^a^ If no interaction between the group (control vs burnout) and the CpG was observed, the main effect of the group (control *vs.* burnout) over all CpGs was reported

^b^ If we observed a significant interaction between the group (control *vs.* burnout) and the CpG, the results were reported for each CpG site separately

**Supplementary table 2.** Correlation between symptoms of burnout and depression with DNA methylation and sBDNF

Correlation between burnout and depressive symptoms and DNA methylation were analysed using a mixed model and association between burnout and depressive symptoms and sBDNF levels were assessed using linear regression analysis. Data are expressed as B-coefficient and confidence intervals (CI).

|  | Exhaustion (MBI-GS) | Cynicism  (MBI-GS) | Professional efficacy (MBI-GS) | Depression (BDI-II) |
| --- | --- | --- | --- | --- |
|  | B (CI)  *p*-value | B (CI)  *p*-value | B (CI)  *p*-value | B (CI)  *p*-value |
| DNA methylaition (%) |  |  |  |  |
| Promoter exon Ia  All CpGs^a^ | 0.06 (-0.01, 0.13)  0.089 | 0.06 (-0.01, 0.14)  0.103 | 0.07 (-0.05, 0.19)  0.245 | 0.01 (-0.002, 0.02)  0.088 |
| Promoter exon Ib  All CpGs^a^ | 0.14 (0.08, 0.21)  **<0.001** | 0.15 (0.08, 0.22)  **<0.001** | -0.12 (-0.24, -0.01)  **0.034** | 0.02 (0.009, 0.03)  **<0.001** |
| Promoter exon IV  CpG1^b^ | 0.16 (0.01, 0.32)  **0.032** | 0.23 (0.07, 0.39)  **0.004** | -0.20 (-0.47, 0.07)  0.14 | 0.03 (0.003, 0.05)  **0.030** |
| Promoter exon IV  CpG2^b^ | 0.05 (-0.03, 0.14)  0.211 | 0.05 (-0.04, 0.14)  0.308 | -0.08 (-0.23, 0.07)  0.308 | 0.003 (-0.01, 0.02)  0.669 |
| Promoter exon IV  CpG3^b^ | -0.03 (-0.10, 0.05)  0.483 | -0.02 (-0.10, 0.06)  0.622 | -0.01 (-0.14, 0.12)  0.868 | -0.001 (-0.01, 0.01)  0.910 |
| Promoter exon IV  CpG4^b^ | - 1. (-0.07, 0.10)   0.807 | -0.04 (-0.13, 0.05)  0.416 | 0.06 (-0.09, 0.21)  0.428 | -0.004 (-0.02, 0.01)  0.560 |
| Promoter exon IV  CpG5^b^ | 0.06 (-0.05, 0.18)  0.295 | 0.12 (-0.01, 0.24)  0.065 | -0.04 (-0.24, 0.17)  0.719 | 0.008 (-0.01, 0.03)  0.450 |
| Promoter exon IV  CpG6^b^ | -0.003 (-0.07, 0.06)  0.924 | -0.02 (-0.09, 0.05)  0.573 | 0.05 (-0.05, 0.15)  0.322 | 0.0 (-0.01, 0.01)  0.967 |
| Promoter exon IV  CpG7^b^ | 0.26 (0.07, 0.44)  **0.008** | 0.22 (0.01, 0.42)  **0.038** | -0.53 (-0.85, -0.21)  **0.001** | 0.04 (0.006, 0.07)  **0.02** |
| Coding region of exon IX  All CpGs^a^ | 0.34 (-0.11, 0.79)  0.138 | 0.15 (-0.34, 0.63)  0.554 | -0.36 (-1.13, 0.41)  0.361 | 0.05 (-0.22, 0.13)  0.168 |
| sBDNF protein expression (ng/mL) | -0.77 (-1.82, 0.28)  0.149 | -0.84 (-1.98, 0.29)  0.144 | 0.61 (-1.20, 2.43)  0.503 | -0.26 (-0.43, -0.08)  **0.004** |

^a^ If no interaction between the group (control vs burnout) and the CpG was observed, the main effect of the group (control *vs.* burnout) over all CpGs was reported

^b^ If we observed a significant interaction between the group (control *vs.* burnout) and the CpG, the results were reported for each CpG site separately
